# Milk as a Factor in the Causation of Disease

**Published:** 1896-05

**Authors:** John M. Parker

**Affiliations:** Haverhill, Mass.


### MILK AS A FACTOR IN THE CAUSATION OF DISEASE.

By JOHN M. PARKER, D.V.S.,
HAVERHILL, MASS.

(Concluded from page 258.)

Some of the principal pathogenic microörganisms found in milk are those of tuberculosis, typhoid fever, cholera, diphtheria, scarlet fever, pus-cocci, streptococci, pneumococci, etc. The most important of these are probably the specific organisms of tuberculosis, typhoid, cholera, diphtheria, and scarlet fever. The first two have their own peculiarities, which make them specially important: Tubercle bacilli, because they are carried from the cow direct, and come into the milk, when present, by way of the udder; the typhoid fever bacilli, because they multiply rapidly in milk at ordinary temperature, so that once finding their way into milk will multiply rapidly and become a serious source of danger. As a precautionary measure, then, the greatest care should be exercised in using water for washing cans and other utensils to make sure that there is no possibility of contamination from this source.

A most interesting illustration of the way in which typhoid fever is spread by the use of infected milk is reported to have occurred at Marylebone, where a farm-hand was suffering from typhoid fever. A large number of persons taking milk from this farm became sick, the outbreak of fever following from house to house, appearing in those houses supplied from this farm, and missing the intervening houses, so that in a certain street all those families that had been in the habit of getting their milk from this farm were sick, while their neighbors escaped. A case is also reported where, after cans were thoroughly washed with soda and boiling water, they were simply rinsed out before milking with clear cold water from a well. The well afterward proved to be infected, and a large number of persons became sick from this cause.

Cholera, scarlet fever, and diphtheria, as well as other infectious diseases may be communicated in the same way, but the immediate danger is not so great, because these organisms will not, as a rule, multiply in milk, so that there is not the same danger of the rapid spread of the disease. These organisms are all destroyed by heating to 180° F.

The bacillus of tuberculosis stands in a class by itself, both because of its special characteristics and because of the amount of work that has been done on the subject. The greatest diversity of opinion prevails as to the danger of tuberculosis from the use of milk. What, then, are the facts in the case?

We find, to begin with, that deaths from consumption are steadily decreasing. In Massachusetts the percentage of deaths for each 1000 of population from 1883 to 1893, inclusive, has fallen from 31.6 per cent. to 22.7 per cent. In New Hampshire, also, the statistics show a gradual falling off in the number of deaths from phthisis pulmonalis. The same decrease appears to a marked degree in Great Britain. This decrease, of course, has nothing to do with the use of milk, but is due to an increased knowledge of the subject and to improved sanitary conditions generally.

A great deal has been said and written on the question of the infectiousness of milk when the udder is not infected. Now, if we take Prof. Ernst's work on the subject, we find that even Prof. Ernst does not claim that the danger is so very great. He simply says that there are cases that are dangerous even where the udder is not affected; but in judging of the amount of danger it must be remembered that all his cases were picked out by physical examination alone. No tuberculin was used, and it is just as possible to pick out these bad cases now as it was then, so that really no milk from such cows as he experimented with should be on the market. In Dr. Ernst's cases thirty-six cows were experimented with, and the bacillus was found in the milk of twelve of these thirty-six.

In the first series of experiments eighty-eight guinea-pigs were inoculated with milk from fifteen of these tuberculous cows, and the milk from only six of these cows was found capable of producing the disease, and only twelve of these eighty-eight guinea-pigs became infected.

In the second series ninety rabbits were inoculated with milk from nineteen different tuberculous cows. The milk from only four of the cows produced the disease, and only six of the ninety rabbits were infected.

In the third series forty-eight rabbits were fed with milk from five tuberculous cows, and only two became diseased, both being fed with milk from the same cow.

Practically, then, when these well-marked cases gave no greater results, it would seem that with the exclusion of these advanced cases the milk would be harmless unless the udder was affected. This seems to have been the opinion of the Royal Commission appointed by the British Government to consider the matter. It is also the opinion of such an able authority as Prof. Theobald Smith, late of the Bureau of Animal Industry. In the *Year-Book of the U. S. Department of Agriculture* he says: "We may, for convenience and clearness, typify those stages: 1. In the earlier stages of the disease, provided the udder is normal, the milk is free from tubercle bacilli. 2. In the more advanced stages, provided the udder is normal, the milk may or may not contain tubercle bacilli. If the disease has become generalized, the indications are that at some time or other tubercle bacilli may pass into the milk. This passage is revealed at autopsy by disease of the glands of the udder. The indications are that this passage is largely temporary, perhaps lasting only a day before the bacilli are caught up and filtered out into the lymphatic system. The indications are, furthermore, that comparatively few bacilli pass through the udder. The udder itself does not favor their development there, and the closest inspection fails to reveal any augmenting foci of disease. These statements are based on careful examination of slaughtered cattle, and the thorough testing of milk from advanced cases."

Practically, then, the danger is confined to cases in which there is disease of the udder; such cases are comparatively rare. There are, I believe, no authoritative statistics showing more than from 2½ to 3½ per cent. of cases of disease of the udder among tuberculous cows. My own experience does not give as high a percentage as that. In Pennsylvania Dr. Ridge gives the percentage of tuberculosis of the udder in the neighborhood of 3 per cent. Dr. Peters gives it as "very small." Dr. Burr gives 12 cows out of 300 cases of tuberculosis as having diseased udders, and Theobald Smith says: "Fortunately tuberculosis of the udder is rare. Among 200 infected animals I have only seen 1 case of udder disease, and 16 others which, according to post-mortem studies, may have shed at one time or another tubercle bacilli into the milk in small numbers, but which had no recognizable disease of the udder itself." He further says: "The large percentage of udder-tuberculosis reported by several writers lately is incompatible with all former statistics, and indicates either an unprecedented condition in certain localities, or else an error in diagnosis." And, finally, in the *Annual Report of the State Board of Cattle Commissioners*, in reports of post-mortem lesions, while disease of the lungs, mediastinal glands, etc., are reported, there is no mention made of even a single case of disease of the udder.

But the best evidence of the absence of great danger is found in such statistics as are collected by Dr. Watson and presented in the *Report of the State Board of Health of New Hampshire* for 1892. These statistics are collected from slaughter-house reports, principally in Germany, and, as Dr. Watson points out, "calves are fed exclusively on cow's milk, so that they offer by far the best of natural experiments as to the amount of danger to human beings from raw cow's milk." He shows that among

| | | | | | |
|---|---|---|---|---|---|
| 23,557 | calves slaughtered | there were | 2 cases | of tuberculosis. | |
| 30,477 | " | " | 1 case | " | |
| 143,218 | " | " | 35 cases | " | |
| 23,592 | " | " | 1 case | " | |
| 800,000 | " | " | 3 cases | " | |
| 24,760 | " | " | 1 case | " | |
| 1,045,604 | " | " | 43, or .0041 per ct. tuberculous. | | |

"The only explanation of these figures," says Dr. Watson, "is that there are few bacilli in the milk of most tuberculous cows."

Taking everything into consideration, then, the only conclusion we can come to is *practically and under natural conditions* the greatest danger from the general use of cow's milk, especially by infants, is the part it plays in the etiology of cholera infantum.

In the mortality report of the *Massachusetts Board of Health for* 1892 it is shown that there were 5739 deaths from phthisis at all ages; 2898 deaths from cholera infantum; 1455 deaths from diphtheria and croup; 827 deaths from typhoid fever; 669 deaths from scarlet fever; 80 deaths from measles. In this report we see cholera infantum placed next to phthisis in the death-rate. Now, in considering this subject, it must be remembered that the latest statistics show that by far the largest proportion of cases of phthisis occur in people who have recently moved into houses previously occupied by consumptives; while, on the other hand, cholera infantum is due almost solely to the ingestion of impure milk. Further, the deaths from phthisis include deaths at all ages, while cholera infantum occurs only in children under five years, and practically under one year old.

In New Hampshire the proportion of deaths from cholera infantum is even greater, for the year ending 1891 the report shows 695 deaths from consumption, and 486 deaths from cholera infantum. The report then remarks "cholera infantum was the cause of more than one-third of all the deaths from prominent zymotic diseases in New Hampshire in 1891."

In Ontario for the year ending 1892, in a population of over 2,000,000, phthisis caused 2592 deaths and cholera infantum 670; but among the 23,190 children under one year old cholera infantum caused 600 deaths, while phthisis caused 283 deaths.

These figures are pregnant with meaning. Cholera infantum, or milk diarrhœa, is purely a disease of infants, due, as a rule, to improper feeding with impure milk. Yet in Massachusetts it was the cause of 2898 out of 11,668 deaths from the six most prominent zymotic diseases. In other words, cholera infantum was the cause of one-fourth of all deaths from all prominent zymotic diseases in Massachusetts in 1892, and of more than one-third of all deaths from all prominent zymotic diseases in New Hampshire in 1891.

Cholera infantum is essentially a preventable disease, and by the simple introduction of better methods of keeping and caring for dairy stock and milk, and by a realization of the absolute importance and necessity of perfect cleanliness, this source of danger can be immeasurably lessened. Knowing what is needed, then, it becomes our duty to spread information on this subject by every means in our power.

If improvements are not introduced, and if advantage is not taken of our increased knowledge on the subject, then the prophesies of certain Western men may come true, and Western Pasteurized milk may enter into serious competition with the dairy industry in the East. It behooves our farmers, then, to brace up and, for their own sake, do what they can to improve the purity and keeping quality of our milk-supply.

